# Additively Manufactured Commercial Co-Cr Dental Alloys: Comparison of Microstructure and Mechanical Properties

**DOI:** 10.3390/ma14237350

**Published:** 2021-11-30

**Authors:** Dalibor Viderščak, Zdravko Schauperl, Sanja Šolić, Amir Ćatić, Matjaž Godec, Aleksandra Kocijan, Irena Paulin, Črtomir Donik

**Affiliations:** 1 Faculty of Mechanical Engineering and Naval Architecture, University of Zagreb, Ivana Lučića 5, 10000 Zagreb, Croatia; zdravko.schauperl@fsb.hr; 2 Department of Mechanical Engineering, University North, Jurja Križanića 31b, 42000 Varaždin, Croatia; ssolic@unin.hr; 3 School of Dental Medicine, University of Zagreb, Gundulićeva 5, 10000 Zagreb, Croatia; catic@sfzg.hr; 4 Institute of Metals and Technology, Lepi pot 11, 1000 Ljubljana, Slovenia; matjaz.godec@imt.si (M.G.); Aleksandra.kocijan@imt.si (A.K.); irena.paulin@imt.si (I.P.); crtomir.donik@imt.si (Č.D.)

**Keywords:** Co-Cr dental alloys, LPBF, microstructure, mechanical properties, SEM, EBSD

## Abstract

Laser-powder bed fusion (LPBF) is one of the preferred techniques for producing Co-Cr metal structures for dental prosthodontic appliances. However, there is generally insufficient information about material properties related to the production process and parameters. This study was conducted on samples produced from three different commercially available Co-Cr dental alloys produced on three different LPBF machines. Identically prepared samples were used for tensile, three-point bending, and toughness tests. Light microscopy (LM), scanning electron microscopy (SEM), and electron backscatter diffraction (EBSD) analyses of microstructure were performed after testing. Differences were observed in microstructures, which reflected statistically significant differences in mechanical properties (one-way analysis of variance (ANOVA) and Scheffé post hoc test (α = 0.05)). The material produced on the 3D Systems DMP Dental 100 had 24 times greater elongation *ε* than the material produced on the Sysma MySint 100 device and the EOS M100 machine. On the other hand, the material produced on the EOS M100 had significantly higher hardness (HV0.2) than the other two produced materials. However, the microstructure of the Sysma specimens with its morphology deviates considerably from the studied group. LPBF-prepared Co-Cr dental alloys demonstrated significant differences in their microstructures and, consequently, mechanical properties.

## 1. Introduction

Additively manufactured (AM) Co-Cr-based alloys are used for metallic structures of dental appliances and generally for biomedical purposes. Due to their excellent mechanical properties with a combination of high wear, corrosion resistance, and biocompatibility, Co-Cr alloys are one of the standard materials in biomedical applications. In addition, Co-Cr alloys for dental appliances demonstrate optimal manufacturing, biomedical, and economic characteristics [1,2].

Laser-powder bed fusion (LPBF) machines for Co-Cr dental alloys are widely available on the market. All manufacturers declare that products produced on their devices meet all professional standards (primarily EN ISO 22674:2016) and are comparable or better to products made by conventional production processing (casting) [3]. However, just a few studies are available that investigate the impact of the production parameters and machine set-up for AM production on the quality and mechanical characteristics of dental metal-based constructions for fixed partial dentures (FPDs) [4,5].

Co-Cr dental alloys are produced from LPBF techniques such as selective laser melting (SLM), which allow us to overcome the imperfections of traditional manufacturing processes (casting and milling). With SLM, layer by layer of metal powder is melted into a 3D object with a directed laser beam. The main advantage of the SLM manufacturing process over traditional casting is the possibility of producing complex personalized objects much faster with similar or better properties compared to conventional manufacturing [5,6,7]. Digital technologies have been in use for more than three decades in dental medicine, with the first CEREC machine utilizing CAD/CAM principles having been on the market since 1986. However, only in the last decade, the exponential development of digital technologies, specifically AM and intraoral 3D scanning devices, has significantly increased their impact and use in everyday clinical practice [6,7,8,9]. This increase followed technological advances in intraoral scanners’ precision, handling, ease of use, compatibility, and interconnectivity with commonly used dental design software such as ExoCAD (ExoCAD Int., San Jose, CA, USA), Trios 3Shape (Trios, Denmark), and InLine (Dentsply-Sirona Co., Charlotte, NC, USA). Clinical implementation of new technologies calls for modifications and optimizations of clinical and technical protocols [10,11]. Within technical protocols are also those dealing with the production of dental prosthodontic appliances regarding input material quality and characteristics, input material handling protocols, production specifics, and output quality control with final standardized products regarding mechanical and biochemical properties, biopotentials, cytological dynamics, and forensic aspects [12,13]. However, those technical production protocols are still undefined and are by no means standardized, resulting in a vast array of products with undefined characteristics. Such a situation on the dental market leaves both dentists and patients unaware of the material quality or the quality of the prosthodontic appliances placed in function, which can have a significant impact on clinical performance and durability as well as potential warranty issues [14,15,16,17,18,19].

Dental and generally biomedical Co-Cr alloys consist of 51.8–65.8 wt % of Co; 23.7–30.0 wt % of Cr; 4.6–5.6 wt % of Mo; 4.9–5.9 wt % W; and <1 wt % of Mn, Si, and Fe [12]. The mechanical properties of Co-Cr dental alloys primarily depend on the chemical composition, microstructure, and manufacturing process. Pure Co exists in two allotropic modifications: at temperatures below 417 °C in the HCP structure, while an FCC structure is stable above this temperature. The microstructure of dental alloys consists of the γ phase (FCC lattice) with carbides and the *ε* phase (HCP lattice). The γ phase affects mechanical properties (ductility) while the *ε* phase affects tribo-corrosion properties (wear and corrosion resistance) [1,20,21,22,23,24]. Properties of Co-Cr dental alloys greatly depend on the γ–*ε* ratio (FCC–HCP ratio) and other inclusions, predominantly carbides, in the microstructure (type, distribution, and quantity of the carbides) [5,25,26,27].

High heat and cooling rates are characteristics of the SLM process. High heat and cooling rates lead to phase transformations and result in microstructural changes in the γ–*ε* ratio, and, consequently, in the material properties (mechanical, wear, and corrosion) of the final products [15,20]. SLM main process parameters such as laser power, scanning speed, layer thickness, hatching distance, the temperature of the building platform, and scanning strategy lead to differences in the microstructure of the produced parts and modifications of the mechanical properties [3,14,26]. Recent studies reported that Co-Cr dental alloys produced by the SLM process have a higher corrosion resistance [26] and higher yield and tensile strengths compared to the cast alloys. In the SLM Co-Cr dental alloys, mechanical anisotropy was detected due to changes in the obtained microstructure [16]. The SLM-produced Co-Cr alloys met the properties according to the ISO 22674:2016 (type 5 criteria) such as UTS, yield strength, and elongation and were higher than manufactured by casting [1,2,3,27,28,29,30,31]. Therefore, optimizing the main SLM production parameters is essential for producing Co-Cr dental alloys with the necessary characteristics for dental appliances [3,26].

The main objective of this study is to compare the mechanical properties of additively produced commercial Co-Cr alloys for dental appliances on three different machines with three types of metal powders where manufacturers guarantee compliance with the requirements according to EN ISO 22674:2016. Three types of samples were additively manufactured (LPBF) for testing mechanical properties and analysis microstructures using light microscopy (LM), scanning electron microscopy (SEM), electronic backscattering techniques (EBSD), and chemical composition analysis to try to correlate these obtained mechanical properties with the microstructure. Based on all these results, we propose the standardization of LPBF procedures for dental use.

## 2. Materials and Methods

**Figure 1:**
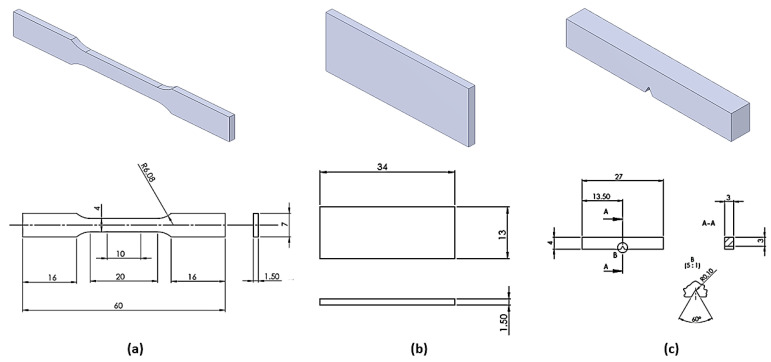
Schemes of the produced specimens: (**a**) static tensile test, (**b**) three-point bending, (**c**) impact toughness (V-notch).

Three sets of three specimens were produced using (a) Sysma MySint100 (BEGO Medical GmbH, Bremen, Germany), (b) EOS M100 (EOS GmbH, Krailling, Germany), and (c) 3D Systems DMP Dental 100 (3D Systems, Rock Hill, SC, USA). All specimens were produced and heat-treated for stabilization according to the producer-recommended parameters. The shape and dimensions of the printed specimens are shown in Figure 1.

We conducted tests for static tensile strength (tensile strength *R_m_*, MPa, and elongation *ε*, %; Beta 50-5, Messphysik, Austria), three-point bending (flexural strength *R_fM_*, MPa; Inspekt Table Blue 20 kN, Hegewald & Peschke Meß- und Prüftechnik GmbH, Germany), Charpy impact toughness test (impact toughness, *CVN*; Charpy impact machine, Karl Frank GmbH, Weinheim-Birkenau, Germany), and microhardness (HV0.2; Wilson–Wolpert Tukon 2100B; Instron, Norwood, MA, USA). The microstructural characterization of the specimens was performed for impact toughness in two vertical cross-sections. For the microstructural analysis, specimens were prepared with the standard metallographic procedure followed by electrochemical etching with 10 vol % of oxalic acid at 12 V for three minutes. The microstructure was analyzed using the light microscope OLYMPUS GX51F-5 with the attached Olympus DP-25 CCD camera. A field-emission scanning electron microscope (FE-SEM), ZEISS CrossBeam 550 FIBSEM (Oberkochen, Germany), equipped with an EDAX Hikari Super EBSD camera, was used for the detailed microstructural characterization with EDAX TEAM software. Secondary electron imaging (SEI) and electron backscatter diffraction (EBSD) was carried out on FE-SEM. SE images and EDS analyses were performed using 15 kV accelerating voltage and 2.0–5.0 nA probe current, while EBSD measurements were carried out on 70° tilted samples and 7.0 nA probe current for phase composition. The EBSD characterization was performed on randomly selected field of appx. 500 × 400 μm. Chemical composition was performed on ICP OES, Agilent 5800 (Santa Clara, CA, USA), and carbon content on ELTRA CS 800 (Eltra GmbH, Haan, Germany).

The obtained values of mechanical properties were analyzed using SPSS Version 20 (Illinois, USA). The values between different groups were evaluated with a one-way analysis of variance (ANOVA) and Scheffé post hoc test (α = 0.05).

Table 1 shows the type and the composition of Co-Cr powder used for each device, as specified by the manufacturer.

## 3. Results

The results of the tested mechanical properties of all specimens are presented in Table 2. The values in the table represent the mean value from three measurements (n = 3 per group) and their standard deviations. The mean values of HV0.2 were measurement (n = 10) in two mutually perpendicular directions of Co-Cr specimens.

The obtained chemical composition of specimens is given in Table 3.

The chemical composition from Table 3 matches the declared chemical composition of the manufacturers. For example, the chemical composition of Co-Cr powders Wirobond C+ (Sysma MySint100) and Cobalt Chrome SP2 (EOS M100) is similar. In contrast, Co-Cr powder Laser Form CoCr (B) (3D System DMP Dental 100) does not have a similar amount of W (0.19 wt %). Crucial is C content due to its effect on the mechanical properties (carbides are formed) [5].

The addition of molybdenum (Mo) and wolfram (W) leads to solid solution strengthening and the formation of MoC- and WC-type carbides and Co_3_W and Co_3_Mo intermetallic phase (HCP–*ε* phase). The carbon content (even in a small range) greatly influences mechanical properties (the volume of γ phase increased—higher elongation *ε*). Co-Cr dental alloys consist mainly of γ phase and carbides of the (Cr, Fe, W, Mo)_23_C_6_ (higher *Rp*_0.2_, *R_m_*, and hardness) [5,32,33]. The obtained mechanical properties depend on the γ–*ε* (FCC–HCP) ratio [26,31].

### Microstructure Analysis

**Figure 2:**
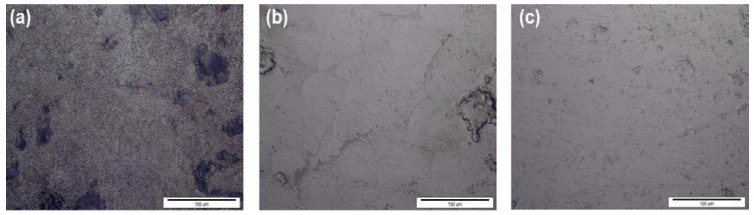
Microstructure, LM, etched: (**a**) Sysma MySint 100, (**b**) EOS M100, (**c**) 3D Systems DMP Dental 100.

**Figure 3:**
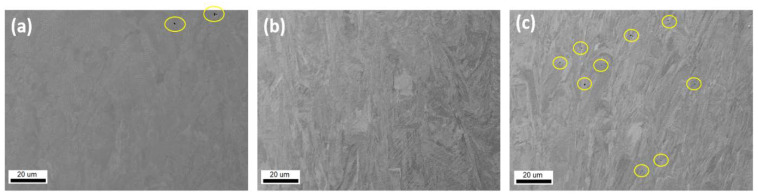
Microstructure of specimens: (**a**) Sysma MySint100, (**b**) EOS M100, (**c**) 3D Systems DMP Dental 100. Yellow circles mark porosities.

The microstructure of the specimens was analyzed using a light microscope. Characteristic microstructures under the same magnification are presented in Figure 2. The microstructure of the specimens built on the Sysma MySint100 device (Figure 2a) [12] indicates morphology of the microstructure, which can be the result of low values of Laser Energy Density (LED) (J mm^−3^) used during the SLM process according to [16]. Low LED values can also lead to large internal porosity and a lack of fusion between the layers [12]. The observed porosity is presented in Figure 3. Figure 2b,c and Figure 3b,c present continuous microstructure of regular morphology, which is characteristic for medium and high values of LED [16].

The SEM analysis shows mostly uniformly annealed microstructure of the specimens with the characteristic morphology for additive manufacturing technologies. Studies of the specimens also show some local porosity (yellow circles in Figure 3). Regardless of the selected areas, no visible differences were observed.

**Figure 4:**
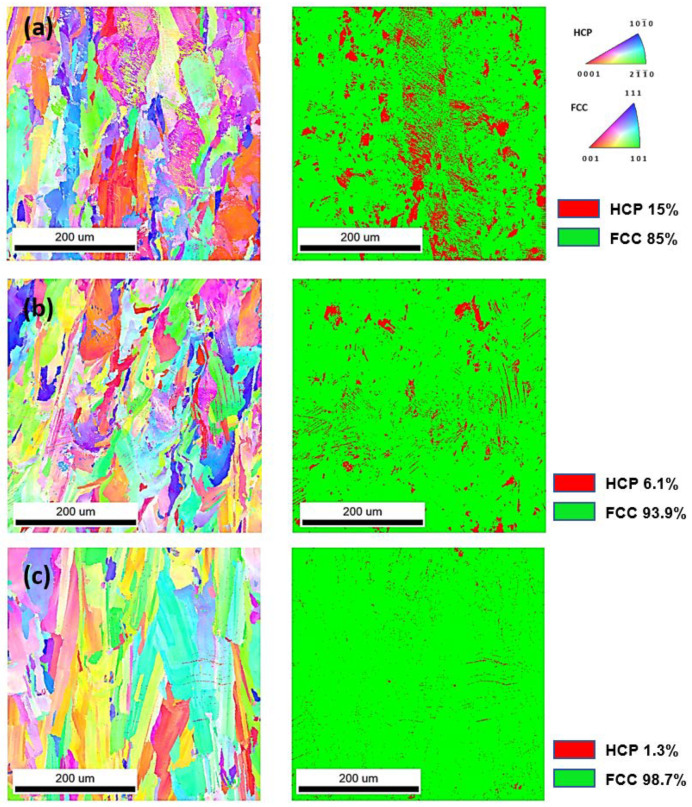
EBSD images of IPF Z with lagend and phase map with the amount of each phase of (**a**) Sysma MySint100, (**b**) EOS M100, (**c**) 3D Systems DMP Dental 100.

The EBSD analyses on prepared samples show the differences in the microstructure. EBSD inversed pole figures in the Z-direction (IPF Z) micrographs and phase maps with HCP and FCC phase amounts are presented in Figure 4. The microstructure of the observed specimens presents typical AM-prepared material with melt pool shapes and cellular structures in different directions. AM processes show that an equiaxed grain shape is formed at a high solidification rate and a low-temperature gradient due to powder thermal conductivity. On the other hand, a planar grain structure is formed with a high-temperature gradient and low solidification, and in between, a columnar grain shape is created [34]. The main differences of the studied Co-Cr samples are in the amount of HCP phase in the prepared sample: 15% in Sysma, 6.1% in EOS, and 1.3% in 3D Systems.

## 4. Discussion

The present study results show significant differences in the values of the mechanical properties of the specimens, depending on the used device shown in Table 2 (*p* values < 0.05 were considered statistically significant).

The most significant deviations are present in elongation *ε*, where specimens made on the 3D Systems DMP Dental 100 have 24 times greater elongation *ε* (0.33 ± 0.1 < 8.1 ± 1.5, *p* < 0.05) than specimens made on the Sysma MySint100 device.

The tensile strength *R_m_* of the specimens produced on EOS M100 has a statistically higher value (*p* < 0.05) from other devices. In contrast, the lowest bending strength is measured on the Sysma MySint100 specimens (*p* < 0.05). Specimens from this device also have the highest standard deviations of *R_m_* and *R_fM_* values.

No differences in microhardness (HV0.2) were measured between the cross and longitudinal sections of the specimens made on the same machines. However, the microhardness of specimens made on the EOS M100 machine is significantly higher than the two other groups of specimens (*p* < 0.05).

Analysis of the microstructures showed that the microstructure of the Sysma MySint100 specimens differ slightly from the other two groups of specimens in its morphology. The microstructure of the other two specimens has a fine-grain microstructure with clearly visible boundaries between the grains. Thus, they have typical morphology for the material obtained by laser melting with melt pools overlapping [23]. The carbide precipitates were not found along the grain boundaries either in the grains.

Based on the literature [3,30,31,35,36,37], the mean values and standard deviations of the mechanical properties of milled (CNC), cast, and SLM-produced Co-Cr alloys, are presented in Table 4, along with EN ISO 22674 criteria.

Comparing the measured values of mechanical properties in Table 2 with the mean values from the literature in Table 4, depending on the production technology, it can be concluded that the SLM technology can produce Co-Cr dental alloys that meet the EN ISO 22674:2016 type 5 criteria (*R_p_*_0.2_ ≥ 500 MPa and *ε* ≥ 2%). They also have better mechanical properties than Co-Cr alloys produced by conventional production processes (milled (CNC) and cast). However, the mean value of elongation *ε* (0.33 ± 0.1) on Sysma Mysint100 does not meet EN ISO 22674:2016 standards and is not comparable with mean values obtained with conventional production procedures (Table 4). On the other hand, other values of all mechanical properties of the materials produced on three different machines from Table 2 are comparable to the obtained mechanical properties in [3,30,31,35,36,37] (Table 4). Therefore, comparing the mechanical properties of specimens prepared by different machines, the 3D Systems DMP Dental 100 can be compared to the values presented in Table 4. At the same time, EOS M100 has an elongation *ε* about 50% lower (4.90 ± 1.1 < 8 ± 1.5, *p* < 0.05) but meets EN ISO 22674:2016. In contrast, specimens obtained with Sysma MySint100 did not meet the elongation value *ε* (0.33 ± 0.1) according to EN ISO 22674:2016.

According to the Co-Cr binary phase diagram, chromium stabilizes the HCP cobalt in conventionally produced material. Nevertheless, in our study, the phase composition of Sysma MySint 100 and EOS M100 produced materials with similar Cr content displays the different amounts of HCP structure. On the other hand, the 3D Systems DMP Dental 100 with the highest amount of Cr contain almost no HCP structure. This phenomenon can be explained by non-equilibrium (rapid) solidification in AM processes. Therefore, it is necessary to perform further microstructural analyzes and mechanical tests of AM-produced samples to determine the mechanisms of microstructural formation.

## 5. Conclusions

From the obtained results, the following conclusions can be drawn:

- The mechanical properties of Co-Cr specimens made on Sysma MySint 100, EOS M100, and 3D Systems DMP Dental 100 are comparable or even better than the mechanical properties of Co-Cr specimens produced by the conventional routes, which are casting and milling (CNC) technologies (except for elongation *ε*, on Sysma MySint 100 and EOS M100).
- The results of the current study demonstrate that the tested specimens meet at least the type 5 criteria in mechanical properties according to the EN ISO 22674:2016 (R*_p0.2_* ≥ 500 MPa and *ε* ≥ 2%), except the Sysma Mysint100 specimens for elongation *ε*.
- The highest elongation *ε* (8.1 ± 1.5%), flexural strength *R_fM_* (2548 ± 54.3 MPa), and toughness CVN (0.61 ± 0.01 J) have specimens produced by 3D Systems DMP Dental 100 (highest content of Cr (29.1 ± 0.2 wt %) and C (0.015 ± 0.002 wt %) without higher content of W (0.19 ± 0.01 wt %) leads to formatting more Cr_23_C_6_ carbides, which determine the higher proportion of γ phase (FCC) and better mechanical properties).
- The highest tensile strength *R_m_* (1370 ± 13.6 MPa), yield strength *R_p_*_0.2_ (1370 ± 13.6 Mpa), and the microhardness HV0.2 in cross-section (770 ± 80) and longitudinal-section (719 ± 59) have the specimens made on the EOS M100 (the higher HCP (*ε* phase) amount contributes to higher hardness and mechanical properties such as *R_m_* and *R_p0.2_* but decreases elongation *ε*–W (5.6 ± 0.09 wt %) and Mo (5.3 ± 0.08 wt %), leading to formatting Co_3_W and Co_3_Mo intermetallic HCP phase).
- Microstructural analyses show the highest HCP amount of 15% (*ε* phase) in Sysma MySint100 specimens (higher content of C (0.008 ± 0.001 wt %) leads to formatting Cr_7_C_3_ (*ε* phase), MoC, and WC carbides, which leads to decreasing mechanical properties (especially elongation *ε*), which causes lower mechanical properties compared to the other two AM-produced materials).

From the obtained results’ deviation among commercial Co-Cr dental alloys producers, the standardization of process parameters and feedstock powders for Co-Cr dental prosthodontic appliances is essential.

## Figures and Tables

**Table 1:** SLM machine, Co-Cr powder type, and chemical composition (wt %) according to manufacturers.

Machine	Co-Cr Powder Type	Powder Chemical Composition (wt %)
Sysma MySint100	Wirobond C+	Co (63.9), Cr (24.7), W (5.4), Mo (5.0), Si (<1.0)
EOS M100	Cobalt Chrome SP2	Co (63.8), Cr (24.7), Mo (5.1), W (5.4), Si (1), other (<1.0)
3D Systems DMP Dental 100	Laser Form CoCr (B)	Co (bal.), Cr (28.0–30.0), Mo (5.0–6.0), Si (0.0–1.0), Mn (0.0–1.0), Fe (0.0–0.5), C (0.0–0.02)

**Table 2:** Mean values and standard deviations of conducted mechanical properties.

Type of Mechanical Test	Mechanical Property		Sysma MySint 100	EOS M100	3D Systems DMP Dental 100
Static tensile test	Elongation *ε*/%		0.33 ± 0.1 ^a^	4.90 ± 1.1 ^b^	8.1 ± 1.5 ^c^
	Tensile Strength *R_m_*/MPa		1112 ± 123.7 ^a^	1370 ± 13.6 ^b^	1016 ± 61.7 ^a^
	Yield Strength *R_p_*_0.2_/MPa		834 ± 62 ^a^	1061 ± 21 ^b^	822 ± 43 ^a^
3-point bending test	*R_fM_*/MPa		2059 ± 251.9 ^a^	2527 ± 92.7 ^b^	2548 ± 54.3 ^b^
(Impact) Toughness	CVN/J		0.26 ± 0.02 ^a^	0.27 ± 0.03 ^a^	0.61 ± 0.01 ^b^
Microhardness	HV0.2	Cross-section	568 ± 27 ^a^	770 ± 80 ^b^	513 ± 12 ^a^
		Longitudinal-section	554 ± 8 ^a^	719 ± 59 ^b^	587 ± 44 ^a^

Different superscript letter in a row indicates statistically significant difference (*p* < 0.05).

**Table 3:** Chemical composition of specimens (wt %).

Machine	Chemical Composition (wt %)
Sysma MySint100	Co (bal.), Cr (24.6 ± 0.2), W (5.7 ± 0.08), Mo (5.3 ± 0.08), Fe (0.19 ± 0.01), Mn (0.05 ± 0.002), C (0.008 ± 0.001)
EOS M100	Co (bal.), Cr (24.8 ± 0.2), W (5.6 ± 0.09), Mo (5.3 ± 0.08), Fe (0.05 ± 0.003), Mn (< 0.01), C (0.005 ± 0.001)
3D Systems DMP Dental 100	Co (bal.), Cr (29.1 ± 0.2), W (0.19 ± 0.02), Mo (5.3 ± 0.2), Fe (0.19 ± 0.01), Mn (0.71 ± 0.02), C (0.015 ± 0.002)

**Table 4:** Mean values and standard deviations of mechanical properties of milled (CNC), cast, and SLM-produced Co-Cr alloys from the literature, and EN ISO 22674:2016.

Property	Milled (CNC)	Cast	SLM	EN ISO 22674:2016
Tensile Strength *R_m_*/MPa	638 ± 25 [31] 1069 ± 10 [3]	450 [30] 520 ± 30 [31] 783 ± 32 [3]	1072 ± 18 [31] 1158 ± 10 [3] 1200 ± 24 [36]	-
Yield Strength *R_p_*_0.2_/MPa	495 ± 20 [31] 672 ± 4 [3]	581 ± 16 [3] 655 [37] 658 ± 44 [31]	783 ± 15 [3] 790 ± 11 [31] 870 ± 26 [35]	≥500
Elongation *ε/*%	10 ± 1 [3] 11.1 ± 1 [31]	8 [37] 8 ± 0.4 [31] 12 ± 2 [3]	8.7 ± 1.06 [36] 12.7 ± 1.9 [31] 13 ± 1 [3]	≥2
3-point bending test *R_fM_*/MPa	-	1136 ± 1 [30]	2501 ± 9.7 [30]	-
Hardness HV10	264 ± 11 [35] 325 ± 18 [31] 353 ± 6 [3]	270 ± 16 [35] 303 ± 15 [3] 324 ± 27 [31]	399 ± 24 [3] 466 ± 13 [35] 475 ± 10 [31]	-

## Data Availability

The data presented in this study are available on request from the corresponding author.
